# A Krukenberg Tumor from an Occult Intramucosal Gastric Carcinoma Identified during an Autopsy

**DOI:** 10.1155/2014/797429

**Published:** 2014-10-16

**Authors:** Yoshiaki Nakamura, Ayako Hiramatsu, Takafumi Koyama, Yu Oyama, Ayuko Tanaka, Koichi Honma

**Affiliations:** ^1^Department of Oncology, Kameda Medical Center, Kamogawa, Chiba 296-8602, Japan; ^2^Department of Gastroenterology and Gastrointestinal Oncology, National Cancer Center Hospital East, Kashiwa, Chiba 277-8577, Japan; ^3^Department of Gynecology, Kameda Medical Center, Kamogawa, Chiba 296-8602, Japan; ^4^Department of Anatomic and Diagnostic Pathology, Dokkyo Medical University School of Medicine, Shimotsuga, Tochigi 321-0293, Japan

## Abstract

A healthy 45-year-old Japanese female developed right pleural effusion, ascites, and a pelvic mass. Bilateral salpingo-oophorectomy resolved the pleural effusion and ascites. Histopathological examination of the ovaries showed bilateral Krukenberg tumors with signet-ring cell carcinoma (SRCC). Extensive testing including upper and lower gastrointestinal endoscopy and whole-body imaging did not detect the primary lesion. Six months after bilateral salpingo-oophorectomy, the patient developed multiple osteoblastic bone lesions in the spine, pelvis, and femurs. A biopsy of the bone marrow showed SRCC. We administered four cycles of S-1 and cisplatin, resulting in the shrinkage of osteoblastic lesions; she remained stable for a year. Then, she developed disseminated intravascular coagulation with disease progression in the bones. Although she was treated with paclitaxel, the disseminated intravascular coagulation progressed, and she died in a month. During the autopsy, microscopic examination revealed four foci of intramucosal gastric SRCC and healthy macroscopic gastric mucosa.

## 1. Introduction

Metastatic tumors in the ovary are common and account for approximately 7–21% of all malignant ovarian tumors [1–3]. A Krukenberg tumor generally refers to a metastatic carcinoma of the ovary and is characterized by the presence of mucin-filled signet-ring cells accounting for at least 10% of the tumor [4]. According to a study of a large series of Krukenberg tumors, the primary tumors comprise gastric (76%), colorectal (11%), breast (4%), biliary tract (3%), and others (6%) [5]. Because the management and prognosis vary depending on the primary tumor [6–10], identifying the primary lesion is important. However, in many cases, the primary tumor cannot be found until the diagnosis of a Krukenberg tumor, and, occasionally, it is never found. Such cases are diagnosed with a “primary Krukenberg tumor” [11]. When the primary tumor is not found, the distinction between a metastatic tumor from an occult cancer and a primary Krukenberg tumor is challenging. In this case, the treatment is guided by histopathological findings.

We describe a patient with a metastatic Krukenberg tumor caused by an occult primary gastric cancer that was found during an autopsy.

## 2. Case Presentation

A 45-year-old Japanese female, who was a healthy asymptomatic hepatitis B virus carrier, visited our hospital in July 2011 complaining of a lower abdominal mass. She was not taking any medications and had no known allergies to medications. Her father had had colon cancer.

**Figure 1:**
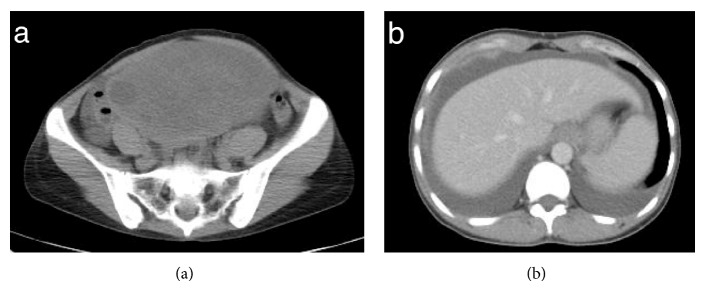
Preoperative computed tomography (CT) findings. (a) CT of the pelvis shows a left ovarian tumor 16 cm in diameter. (b) Bilateral pleural effusion and ascites.

On physical examination, she appeared as a healthy thin female. Her weight was 43.3 kg, blood pressure 94/60 mmHg, pulse rate 70 beats per minute, respiration rate 18 breaths per minute, and body temperature 36.5°C. There was a large nontender palpable mass in the lower abdomen. The laboratory findings were unremarkable with the exception of an elevated CA125 level (117 U/mL). CEA (2.1 ng/mL) and CA19-9 level (14.3 U/mL) were normal. Computed tomography (CT) of the chest, abdomen, and pelvis showed a 28 × 22 × 5 cm left ovarian mass, right pleural effusion, and ascites (Figures 1(a) and 1(b)).

**Figure 2:**
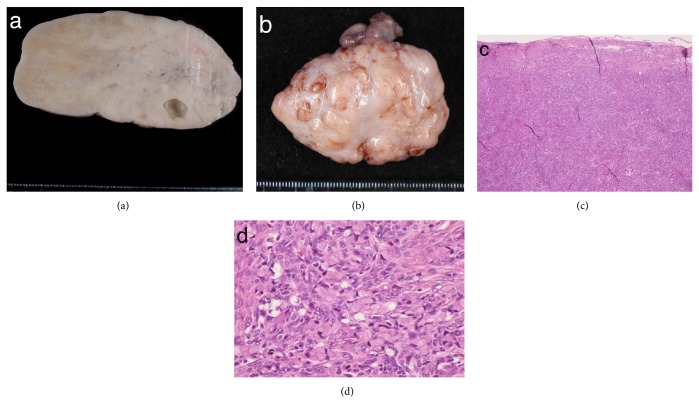
Macroscopic and microscopic findings in the resected ovarian tumors. (a) Left tumor. (b) Right tumor. (c) Microscopic view of the main tumor (hematoxylin and eosin staining (H&E), ×100). (d) Invasive proliferation of signet-ring cells; the desmoplastic stroma (H&E, ×200).

A tentative diagnosis of Meigs' syndrome was made, and she underwent bilateral salpingo-oophorectomy (BSO) with resolution of the pleural effusion and ascites. On histopathological examination of the resected specimen, the left and right ovaries were 15.5 × 12 × 8 cm and 5.5 × 4.5 × 3.5 cm, respectively (Figures 2(a) and 2(b)). Results of the microscopic analysis of both ovaries showed invasive proliferation of a signet-ring cell carcinoma (SRCC) with prominent lymphovascular invasion within the desmoplastic stroma (Figures 2(c) and 2(d)). Immunohistochemical analysis revealed that these tumor cells tested positive for CK7, CK20 (weakly), MUC5AC, MUC6, CDX2 (patchy), CEA, and CA19-9 and negative for MUC2, ER, and PgR.

**Figure 3:**
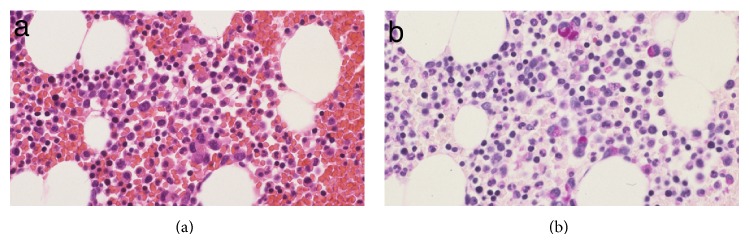
Microscopic findings in the bone marrow. (a) Hematoxylin and eosin staining (H&E), ×200. (b) PAS staining reveals infiltration by signet-ring cells.

Although the histopathological findings were consistent with metastases from a gastric adenocarcinoma, there were no signs of a gastric cancer. The repeat esophago-gastro-duodenal endoscopy only showed atrophic gastric mucosa, but there were no further mucosal aberrations that would be suggestive of early gastric cancer or any lesions suggestive of advanced gastric cancer. A random biopsy showed only chronic gastritis without tumor cells. To rule out any other possible malignant tumors, we performed multiple tests as follows: colonoscopy, magnetic resonance imaging (MRI) and CT of a thin slice of the pancreas and biliary tree, breast ultrasonography, and breast MRI, but the results were all negative. The patient was under observation without systemic treatment. Six months after BSO, she developed asymptomatic multiple osteoblastic lesions on the vertebrae, pelvic bone, and bilateral femurs. A bone marrow biopsy showed scattered signet-ring cell infiltration (Figures 3(a) and 3(b)).

We treated her with a standard Japanese gastric cancer regimen with cisplatin (60 mg/m^2^ on day 8) and S-1 (80 mg/m^2^ on day 1–21) for four cycles, and those osteoblastic lesions mildly shrunk. After approximately a year of stable disease, she developed severe back pain. Laboratory tests showed a decreased hemoglobin level (7.6 g/dL), platelet number (4.2 × 10^4^/*μ*L), and fibrinogen level (43 mg/dL). There were schistocytes on a peripheral blood smear. Prothrombin time (INR 1.62) and activated partial thromboplastin time (38.9 s) were prolonged, and the D-dimer level (>36.0 *μ*g/mL) was elevated. The plasmin-*α*2-plasmin inhibitor complex (20.4 *μ*g/mL), thrombin-antithrombin complex (>60.0 ng/mL), and lactate dehydrogenase (814 IU/L) levels were elevated substantially. CT revealed progression of multiple bone metastases. She was diagnosed with disseminated intravascular coagulation (DIC) with progression of the carcinoma. She was treated with paclitaxel (80 mg/m^2^ weekly) as second-line and blood product support but died in a month with the progression of DIC.

**Figure 4:**
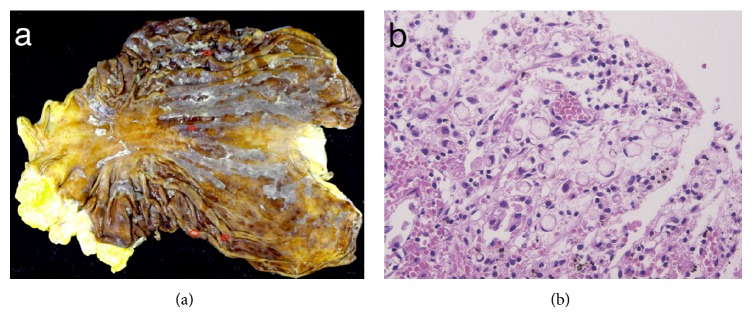
Macroscopic and microscopic findings in the stomach material during the autopsy. (a) Macroscopic analysis of the stomach shows no tumor; red rings indicate the cancerous lesions detected microscopically. (b) Microscopic findings; signet-ring cells exist only on the surface in the mucosa.

The autopsy revealed the following. On macroscopic examination, the gastric mucosa was erosive, but there was no tumor (Figure 4(a)). Nevertheless, with an extensive meticulous microscopic search (sections prepared from 89 blocks) of the stomach, we were able to detect four small foci of SRCC within the atrophic mucosa in the greater and lesser curvature of the lower gastric body (Figure 4(b)). Diameters of all four lesions were smaller than 1 mm. Although lymphatic invasion was not seen, lymphatic vessels were found near the surface epithelium. Immunohistochemical analysis (IHC) revealed that those tumor cells tested positive for CK7, CK20 (weakly), MUC5AC, CEA, and CA19-9 and negative for MUC2, MUC6, and CDX2. The bone marrow of the vertebral column, sternum, and iliac bone was extensively affected by the cancer. There were also widespread lymph node metastases in Virchow's, pulmonary hilar, and perigastric, peripancreatic, and retroperitoneal regions. There were lymphangitic metastases in the lung and tumor cell infiltration of the uterine cervix with dilation of bilateral cardiac ventricles, liver congestion, bone marrow necrosis, multiple hemorrhagic erosions of the entire gastrointestinal tract, diffuse alveolar damage of the lungs, and swelling of the kidney. These findings were indicative of congestive heart failure, shock, and DIC.

## 3. Discussion

We managed a case of a Krukenberg tumor. We identified intramucosal gastric SRCCs during the autopsy. These small tumors were assumed to be the origin of the Krukenberg tumor because postmortem examination showed no primary tumor other than the one in the stomach. To the best of our knowledge, this is the first report of a Krukenberg tumor managed according to an assumption of the existence of an occult gastric carcinoma, which was subsequently proven during the autopsy.

To identify the primary site, we performed extensive testing, including a repeat esophago-gastro-duodenal endoscopy, but these results were all negative. In fact, the tumors found during the autopsy were considerably small to be detected macroscopically.

Although several methods did not help us to identify the primary site, some features suggested that this case was a metastatic tumor from a primary site in the stomach. The ovarian tumors had some relevant features, such as the signet-ring cell component, bilaterality, a nodular appearance on gross examination, and extensive lymphovascular invasion. One study showed that these characteristics are specific to a metastatic ovarian tumor [12]. In addition, immunohistochemical staining of the ovarian tumor (CDX2, CK7, CK20, MUC5AC, and MUC6 were detected and MUC2 was not detected) favored metastases from a gastric SRCC [13–15]. On the basis of these observations, we decided to manage the disease as a gastric carcinoma.

An intramucosal gastric carcinoma rarely metastasizes, but some reports described a Krukenberg tumor from an early gastric cancer [16, 17]. There is a case report of a Krukenberg tumor caused by a gastric mucosal tumor 3 mm in diameter [5]. Our case involves one of the smallest gastric lesions reported to date as an origin of ovarian metastases. The risk of lymph node metastases in an undifferentiated intramucosal gastric carcinoma is higher than in a differentiated type (4.2% versus 0.4%) [18]. Atrophic gastritis as a complication is also a risk factor of metastases because lymphatic capillaries get closer to the mucosal surface in such patients, and, consequently, the intramucosal cancer cells may infiltrate the lymphatic capillaries more easily [16]. In our case, lymphatic vessels were near the tumor cells close to the surface of the gastric atrophic mucosa. The undifferentiated histological appearance of these gastric carcinomas and atrophy of the background mucosa might raise the probability of metastases.

“Primary Krukenberg tumors” were reviewed by Joshi [11]. An autopsy that did not uncover any primary tumor except for the ovary served as a criterion for the diagnosis of a primary Krukenberg tumor in his review. Just like our case, however, some cases require microscopic examination of full sections of the stomach to find the carcinoma. Previously, Kraus reported a case where the primary site was found only after analysis of slices from more than 200 sections [19]. In Joshi's review, it was not described how the detailed examination was performed to rule out an occult gastric carcinoma in each case. Our case leads us to believe that the diagnosis of a “primary Krukenberg tumor” cannot be made without a detailed autopsy for the purpose of ruling out an occult primary carcinoma.
